# Gender and Power Dynamics of Social Relationships Shape Willingness to Participate in Biomedical HIV Prevention Research Among South African Adolescents and Young Adults

**DOI:** 10.3389/frph.2021.639391

**Published:** 2021-05-20

**Authors:** Kalysha Closson, Laura Lee, Janan J. Dietrich, Mags E. Beksinska, Stefanie Hornschuh, Patricia Smith, Jenni A. Smit, Thumbi Ndung'u, Mark Brockman, Glenda Gray, Angela Kaida

**Affiliations:** ^1^Faculty of Medicine, School of Population and Public Health, University of British Columbia, Vancouver, BC, Canada; ^2^Branch for International Surgical Care, Faculty of Medicine, University of British Columbia, Vancouver, BC, Canada; ^3^Perinatal HIV Research Unit (PHRU), Faculty of Health Sciences, University of the Witwatersrand, Johannesburg, South Africa; ^4^Health Systems Research Unit, South African Medical Research Council, Bellville, South Africa; ^5^Maternal Adolescent and Child Health Research Unit (MRU), Faculty of Health Sciences, University of the Witwatersrand, Durban, South Africa; ^6^Faculty of Health Sciences, Simon Fraser University, Burnaby, BC, Canada; ^7^HIV Pathogenesis Programme and Africa Health Research Institute, University of KwaZulu-Natal, Durban, South Africa; ^8^Ragon Institute of Massachusetts General Hospital, Massachusetts, Institute of Technology and Harvard University, Cambridge, MA, United States; ^9^Max Planck Institute for Infection Biology, Berlin, Germany; ^10^Office of the President, South African Medical Research Council, Cape Town, South Africa

**Keywords:** sexual and reproductive health, young people, South Africa, youth-friendly services, gender, biomedical HIV prevention, socioecological model

## Abstract

**Background:** Understanding young women and men's perceived barriers and facilitators to participation in biomedical HIV prevention research is important for designing youth friendly services (YFS) and acceptable technologies, which are necessary for preventing high sustained HIV incidence in South Africa. This study explores the multileveled barriers and facilitators to young men and women's willingness to participate in hypothetical biomedical HIV prevention research.

**Methods:** Eight age- (16–18 and 19–24 years) and gender-stratified focus group discussions (FGDs) were conducted using semi-structured interview guides to explore young South African women and men's willingness, perceived barriers, and facilitators to participating in biomedical HIV prevention research. FGD transcripts were uploaded to NVivo and coded collaboratively with youth study team members. Thematic analysis using Bronfenbrenner's ecological model (individual, inter-personal, community, and societal) was used to guide a deductive coding procedure, which was documented and compared by gender.

**Results:** Thirty-one participants from Durban and 34 from Soweto participated in FGDs. Individual facilitators for participation were discussed more by young men and included financial incentives and altruism. Concerns about side-effects of biomedical products were a common barrier. Interpersonal relationships with peers, intimate partners and caregivers influenced young people's willingness to participate in HIV prevention research, more so among young women. For young women, gendered power dynamics and distrust of intimate partners and parents influenced both communication regarding participation and willingness to participate in research that is often stigmatized, due to societal norms around women's sexuality. On a societal level, participants expressed distrust in medical and research institutions, however a sense of community that was developed with the study staff of this project, was a motivator to participate in future studies.

**Discussion:** At each level of the ecological model, we found participants expressed gendered barriers and facilitators for participation. Gender norms as well as distrust of partners, parents, and health care professionals were key barriers that cut across all levels. At each level participants discussed facilitators that were youth-engaged, underscoring the need to implement YFS, establish trust and address gender inequities within future biomedical HIV prevention studies wishing to engage and retain South African youth.

## Introduction

In South Africa, the nation with the highest number of people living with HIV (~7 million) worldwide, young women aged 15–24 face the highest HIV incidence, accounting for ~2,000 new infections every week (1). Numerous bio-behavioral strategies exist to effectively prevent HIV transmission including, HIV testing and counseling (2), use of antiretroviral therapy (ART) to achieve viral suppression among people living with HIV (UequalsU), consistent condom use, male medical circumcision (MMC) (3), and pre- and post- exposure prophylaxis (PrEP, PEP) among others (4–6).

Advances in biomedical HIV prevention technologies such as oral daily PrEP have shown efficacy, with protection variation largely explained by differences in PrEP adherence and trial retention (7). Across several implementation trials and demonstration projects, adolescent girls and young women in sub-Saharan Africa, have experienced particular challenges to PrEP uptake, adherence, and retention (7). Thus, while PrEP is highly effective when taken as directed (8), due to many intersecting barriers, young people who have the greatest unmet need for HIV prevention modalities, are not fully benefiting from PrEP programming (7). Similar challenges have been noted for youth participation in HIV vaccine research highlighting that women may feel ambivalent toward participating in HIV prevention research, because of a general mistrust toward clinical trials (9), and that adolescents and young adults in South Africa who are at greater risk of acquiring HIV, may be less willing to participate in HIV vaccine trails (10). While some research has quantitatively examined differences in youth who may be more or less likely to participate in biomedical HIV research, with much of the focus being on HIV vaccine trials (10), there is less of an understanding as to why some young men and women may be more or less willing to participate. As researchers continue to discover, develop, and implement HIV prevention strategies including new PrEP formulations and dosage (e.g., long-acting injectable PrEP (11, 12), vaginal microbicides, vaginal rings) and preventative HIV vaccines, it is paramount that studies are inclusive to the needs of adolescents and young adults (13).

Decision-making about participating in biomedical HIV prevention research is complex (14). Most research on willingness to participate in HIV vaccine studies and other biomedical trials has focused on individual-level barriers and facilitators including personal concerns about PrEP safety and potential side-effects, the personal value of participating in research (e.g., benefits of care received and compensation), and valuing being able to contribute to the enhancement of knowledge (15). Few studies, however, have considered the broader multi-leveled determinants that shape decisions to participate. One Tanzanian-based study highlighted that beyond individual-level determinants, community attitudes toward research and structural impediments including stigma, unemployment and poverty play an important role in research participation willingness (16). Results from qualitative research examining views of HPV vaccination among South African youth and their caregivers found that the decision to vaccinate was youth-led, highlighting high levels of youth autonomy in health-related decision-making (17). Further research is needed to explore how gender intersects with the multi-leveled determinants and factors that influence young men and women's decision to participate in biomedical HIV prevention trials.

The dynamic inter-relations between individual and broader social and environmental factors that shape decision-making are conceptualized in Bronfenbrenner's ecological framework (18). This framework and model acknowledges that any outcome cannot be explained by one factor, but instead is the result of a number of interacting factors at different relational and structural levels; individual, interpersonal, community, and societal/institutional or structural. This framework has been used in adolescent and youth research in numerous global contexts (19), including research on understanding young women's risk of HIV in South Africa (6).

Young people living in today's South Africa are the first generation of youth growing up post-apartheid (1994–current). Despite living their whole lives in the new democratic republic, historical legacies of colonialism and apartheid persist to influence the ways in which young people form identities and relationships (20). For example, in the face of adversity, low employment opportunities, and high levels of poverty, young men who are unable to achieve hegemonic forms of masculinity, may form masculine identities that are more controlling, violent, and in turn increase the risk of HIV among young women (21, 22). Further, the formation of differing forms of femininity, which either are acquiescent to persistent gender inequities or seek to resist current gender norms impact the ways in which young women form relationships (23). This body of literature is situated within Connell's theory of gender and power, which mainly focuses on power dynamics between men and women within intimate relationships and how these dynamics influence decision-making processes that effect the health and well-being of women (24). We seek to extend the theory of gender and power beyond power interactions within male and female intimate relationships. In doing so, we wish to explore the ways in which power and gender intersect at each level of the ecological model to influence young men and women's ability and willingness to participate in biomedical HIV research.

The aim of this qualitative study was to explore the perceived barriers and facilitators to participating in hypothetical HIV biomedical prevention research for young women and men living in Durban and Soweto, South Africa. Using the ecological model, we sought to explore the influence of gender and power dynamics at individual, interpersonal, community, and societal levels on willingness to participate. Results from this study can provide researchers, policy makers, and program developers with the knowledge required to better tailor HIV prevention research programming, to improve youth engagement.

## Methods

### Study Setting and Design

#### The AYAZAZI Study

This was a qualitative sub-study of the AYAZAZI study, a multi-site, interdisciplinary, prospective cohort focused on understanding linked patterns of socio-behavioral and biomedical HIV risk among youth aged 16–24 years in Durban and Soweto, South Africa (25). The Durban cohort was based at the Commercial City site of the MatCH Research Unit (MRU) while the Soweto cohort was based at the Perinatal HIV Research Unit (PHRU) at Chris Hani Baragwanath Hospital. Acknowledging the historical and social-political landscapes of HIV research among youth, the AYAZAZI study was grounded in a youth-engagement approach (26, 27).

Between November 2015 and April 2016, 425 youth (aged 16–24 years; HIV negative or unknown HIV status) were enrolled in AYAZAZI and completed baseline procedures, including a quantitative bio-behavioral questionnaire, and were followed every 6 months until 12 months (Durban) or 18 months (Soweto). Additional details of the AYAZAZI study methodology have been previously reported (25, 28, 29).

### Qualitative Component

#### Participant Recruitment for FGDs

Within 6 months of enrollment, a purposive sample of AYAZAZI participants were invited to participate in this qualitative sub-study. We examined baseline questionnaire responses to “how willing are you to participate biomedical HIV prevention and/or vaccine research studies research” and randomly sampled participants who reported being “willing,” “neutral,” or “not willing” to participate in biomedical HIV prevention research, stratifying by gender and age category (16–18 and 19–24 years). Sampled participants were invited to participate in one of eight age- and gender-stratified FGDs, four in Durban and four in Soweto. Those who were interested and willing to participate in FGDs were invited to the respective study sites to receive additional information about the qualitative sub-study. FGDs occurred in April and May 2015 at the PHRU and in July 2016 at the MRU. Participants were included if they had responded to baseline questions regarding willingness to participate in HIV prevention and/or vaccine research, were residing in Soweto or Durban, and were able to provide written informed consent or parental consent and participant written assent if under the age of 18. Participants were excluded from recruitment if they were currently participating in another clinical or observational HIV prevention study.

#### Data Collection for FGDs

Prior to each FGD, trained youth research assistants led an information session with experienced multi-lingual co-facilitators trained in qualitative research methods on what is involved in participating in HIV prevention research such as HIV vaccine trials, MMC studies, PrEP studies, and other studies requesting collection of blood, urine, vaginal swabs (for female participants), seminal fluids (for male participants), and rectal (males and females) samples. The information sessions were delivered in English and used PowerPoint slides and handouts for the participants. However, the facilitators were able to explain concepts and answer participants' questions in IsiZulu or Sesotho, as preferred by participants. Female facilitators led information sessions for female FGDs, and male facilitators led sessions for male FGDs. Following the information session, the facilitator began the FGD. The FGD guide asked participants about their willingness to participate in different types of biomedical HIV research and the reasons that informed their willingness. FGDs were conducted in English although participants were invited to respond in their preferred language and clarifications were made by facilitators in other languages as needed. FGDs including the information session, lasted from 60 to 90 min. All FGDs were audio-recorded, translated (as necessary), and transcribed. In order to maintain confidentiality, participants chose pseudonyms that were used within the transcripts. De-identified transcripts were uploaded to NVivo 12 for analysis (30).

Socio-demographic characteristics reported during the AYAZAZI baseline survey were linked using participant study IDs to identify and highlight the diversity of the sample of youth who participated in the FGDs. Socio-demographic characteristics included site (Durban, Soweto), age (continuous), sexual orientation (lesbian, gay, or bisexual vs. heterosexual), ever had children (yes vs. no), and formal vs. informal housing (e.g., shack, hostel, reconstruction development program [RDP] housing). Participants responses to the whether they would be willing to participate in a HIV vaccine trial (willing, neutral, unwilling) were also recorded at baseline, and linked to participants in the FGDs.

#### Data Analysis

**Figure 1:**
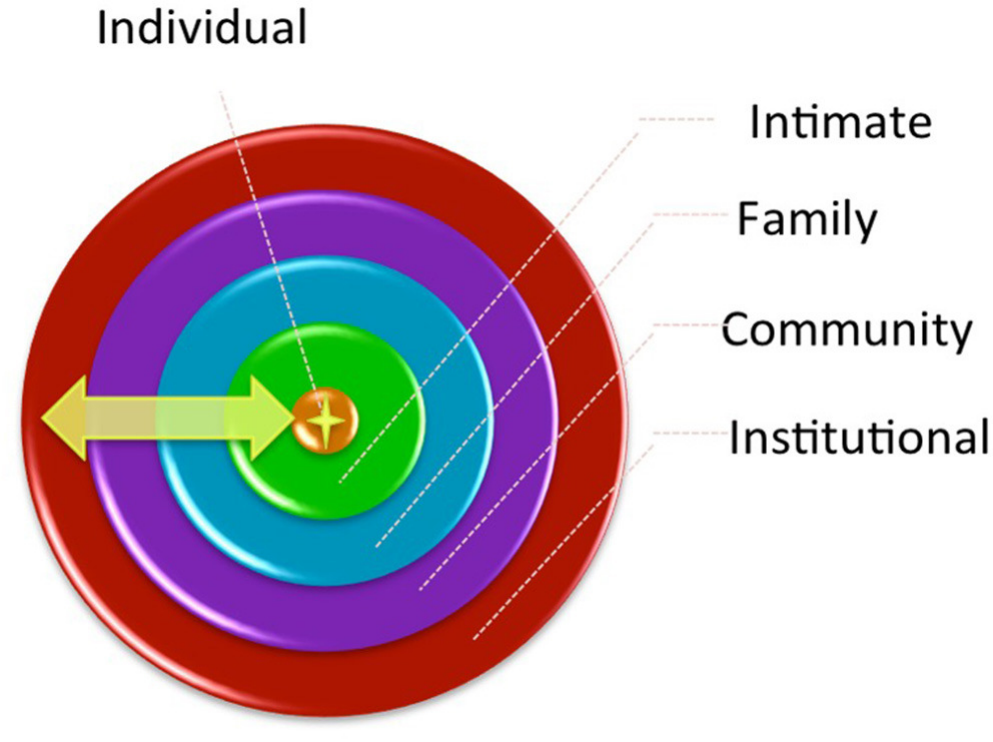
Young women and men's social relationships at various levels of their social environment (18).

To analyze the qualitative data from the FGDs, a team of researchers from Canada and South Africa carried out a collaborative thematic analysis process that prioritized youth engagement. The six Thematic Analysis phases defined by Braun and Clark (31) and Nowell et al. (32) were followed and steps were taken during each phase to ensure trustworthiness (See Table 1). With the goal of gaining a deep understanding of young women and men's views and perspectives on participating in different types of biomedical HIV research, inductive coding was primarily done, as the data was drawn on to determine themes. Some deductive coding was applied later based on the socioecological framework (See Figure 1). Analysis was guided by a latent approach, as the research team read into the subtext in the data and the relational dimensions that were explored by participants. As codes, sub-themes and themes developed, these were verified collaboratively, including with two young researchers who participated in each step of the process. We used participant pseudonyms, chosen by the participants, to share direct quotes from the FGDs that exemplified the sub-themes.

**Table 1:** Ensuring trustworthiness through a collaborative, youth-engaged thematic analysis process.

**Phase of Thematic Analysis**	**Steps to ensure trustworthiness**
1. Familiarization with the data	• The analysis plan was collaboratively developed by team members in Canada and South Africa • Each team member read through all transcripts • A summary was developed for each transcript and reviewed by all team members • Initial reflections about potential codes and themes were recorded
2. Generating initial codes	• A codebook was developed collaboratively by the team, including two youth research assistants • Each code was defined, ensuring input from members from each research site • Regular team meetings were held to discuss the analysis process and encourage reflexivity
3. Searching for themes	• Each transcript was double-coded • Emerging themes were discussed, and interconnected concepts explored • Each transcript was printed out and co-coded line for line by team members and youth research assistants and emerging themes and sub-themes were discussed
4. Reviewing themes	• Transcripts were re-read to verify themes • In weekly team meetings with the global team, the codebook was revised as codes were grouped under one or more themes
5. Defining and naming themes	• Meetings were held with the global team to refine the codebook and to discuss the interpretation and definition of concepts related to themes and sub-themes • The codebook was finalized with codes categorized under themes and sub-themes
6. Producing the report	• The team ensured that the analysis process was described in detail, including reasons for methodological and analytical choices, and that descriptions of context was included in all reports

*Adapted from Nowell et al. (32), p. 4*.

### Ethical Considerations

All participants aged 18–24 years provided voluntary informed consent at enrollment. For participants aged 16–17 years, parents/legal guardians provided voluntary written informed consent and the participant provided voluntary written informed assent. Ethical approval for these procedures was provided by the Office of Research Ethics (ORE) of Simon Fraser University (Canada) and the Human Research Ethics Committee (HREC) of the University of the Witwatersrand (South Africa), and a reciprocity agreement with the University of KwaZulu-Natal Biomedical.

## Results

### Participant Characteristics

In total 65 youth aged 16–24 years (48% young women) participated in the FGDs. Table 2 presents baseline socio-demographic characteristics of the sample overall and stratified by gender. Just over half of participants were from Soweto and 9.2% identified as lesbian, gay or bisexual. At baseline 23% had ever had a child, 75% lived in formal housing, and 71% reported being a student/learner. During the baseline questionnaire when asked if participants of the FGDs would be willing to participate in an HIV vaccine trial 28% indicated they were unwilling, 29% indicated they were neutral, and 43% indicated they were willing. More young women than young men reported being neutral about HIV vaccine participation (39% vs. 21%) and more young men than young women were willing to participate in HIV vaccine trials (50% vs. 36%).

**Table 2:** Characteristics of AYAZAZI participants in focus group discussions (FGDs), overall and by gender (*n* = 65).

**Socio-demographic Characteristics**	**Overall *n* (column%)**	**Young women (*n* = 31) (%)**	**Young men (*n* = 34) (%)**
**Site**			
Durban	31 (47.7)	15 (48.4)	16 (47.1)
Soweto	34 (52.3)	16 (51.6)	18 (52.9)
**Age** median Q1, Q3	18 (17–21)	18 (17–22)	18 (17–20)
**Sexual Orientation**			
Lesbian, Gay, or Bisexual	6 (9.2)	<5	<5
Heterosexual	59 (90.8)	–	–
**Have Children**			
No	50 (77.0)	19 (61.3)	–
Yes	15 (23.1)	12 (38.7)	<5
**Live in Formal Housing**			
No (hostel, reconstruction development project, shack)	16 (24.6)	10 (32.3)	6 (17.7)
Yes	49 (75.4)	21 (67.7)	28 (82.4)
**Willing to participate in HIV vaccine trial?**			
Unwilling	18 (27.7)	8 (25.8)	10 (29.4)
Neutral	19 (29.2)	12 (38.7)	7 (20.6)
Willing	28 (43.1)	11 (35.5)	17 (50.0)

*Percentages by gender are calculated by column and in order to protect participant confidentiality are not displayed if cell count was less than 5*.

### Findings

Throughout the FGDs, youth spoke of barriers and facilitators to participating in biomedical HIV prevention research. Given that young men and women participated in separate FGDs, they were enabled to explore gendered experiences and perspectives, and the barriers and facilitators spoken about were multileveled, occurring on individual, interpersonal, community, and structural lines. Below we present common barriers and facilitators to participation at each level of the ecological model. We highlight and note gender differences at each level and summarize cross-cutting barriers and facilitators that shaped young women and men's willingness to participate in hypothetical biomedical HIV prevention research.

#### Individual

Common individual-level factors that served as facilitators to participation in HIV prevention biomedical studies included opportunities to access food, financial incentives, sexual health information, and other forms of sexual and reproductive health care that are commonly included as part of study visits. Perceived barriers centered around a lack of information about the HIV prevention modality and the fear of side-effects, particularly from PrEP and HIV vaccine candidates.

In the younger-aged female FGDs, participants discussed enjoying being provided reimbursement and something to eat, which they experienced as part of participating in AYAZAZI^1^.

***Phumla***^2^*: “I like the fact that you always give us money when we come here”*.

***Lena**: “I like that you are very honest, and Thaka said food.”*

***Thaka**: “as long as it's good food, good food”*

***Young women, Soweto, 16-18 years FGD***

Another facilitator discussed by young women and men was how research participation facilitated access to knowledge around HIV and helped to provide information about new HIV technologies such as HIV vaccines. For example, Busi in Durban (16–18 years FGD) shared that, “*I would get more knowledge about the HIV vaccine and how it helps us and people, not only young people actually*.”

Participants also expressed how they would want to participate for altruistic purposes in order to benefit their communities.

***Paul**: “I think it will be a benefit for my country and other people related to me because if I take part of the study and the study works out on me, positively so, and maybe the doctors will be able to, through the study and the vaccine, to find a cure or something like that. That…that's what will motivate me.”*

***Young men, Soweto, 16-18 years FGD***

Across all eight FGDs, participants discussed the potential protective effects of participating in HIV prevention research. Beyond the potential for preventing the acquisition of HIV, women in Durban further discussed how participation in studies taking vaginal or rectal swabs could potentially screen for other diseases such as cancer.

Young men were particularly interested in the sexual risk protection benefits to participating in HIV prevention research. They admittedly discussed that at times when they get intoxicated, they may not trust their own decision-making, thus PrEP could protect them if they engaged in behaviors that may expose them to HIV.

***Sipho:*** “*Yes, that PrEP keeps you on the safe side you see. It keeps you on the safe side, when you are drunk, in things like that, you sometimes go to the (night) clubs. In the (night) clubs you cannot be sure that there is not even one person who has the virus in that (night) club. Unfortunately, you will be the one who sleeps with the person who has it. You co-incidentally find that you get someone who has the virus and they won't tell you that. What can make me participate in PrEP (studies) is that it keeps you on the safe side of things, even if something unexpected happens you will have something protecting you. Ja.”*

***Young men, Durban, 16-18 years FGD***

Both young women and men expressed concerns about side-effects and were skeptical of the evidence behind some of the technologies that were being tested within trials. In response to a question asking why you would not take part in an HIV vaccine trial, Thuli in Durban (young women 16–18 years FGD) replied, “*Because they're still not sure of what would it do to your body*.” Young men in Soweto were also curious to know more about the potential side-effects of PrEP.

***Fader**: “I want them to explain to me how it works in the body and assure me that the side-effects won't affect me in future, if I decide to take this pill.”*

***Young men, Soweto, 16-18 years FGD***

Young men were particularly skeptical about participating in studies collecting rectal and sperm samples. Some young men discussed how they would not participate because they had too much ‘pride’, feared the procedure would be painful or uncomfortable, or would be worried that their sperm would be donated, resulting in an unknown child.

***Shaka**: “*Maybe *you can say that its pride, one's pride that is within them, they were born with it, that no you do not want a person to touch you and you too you do not want to put something in a place that was made, not for inserting anything. Ja.”*

***Young men, Durban, 16-18 years FGD***

#### Interpersonal

Interpersonal relationships with peers, intimate partners, and family/caregivers were shown to be important facilitators in shaping young people's willingness to participate in HIV prevention research. These were expressed both as barriers and facilitators.

##### Peers

Stigma and judgment from peers were seen as strong barriers to participation in HIV prevention research, particularly for young men. Some young men were concerned about peers judging their participation in studies involving having to take anal or semen samples. As Sjojo from Soweto (age 19–21) shared,

***Sjojo**: “Eh yha they'll [friends] be this ones I'm going to feel confident about to tell people, like the anal one eh I won't because ah I don't feel comfortable to tell someone else my brother I'll have to yeh you see … something and stuff, then even this one for seminal fluids and stuff I won't feel comfortable to tell someone else that I was masturbating and stuff cause obviously we know how a black person can be like, you know ((laughing)) ((takes it)) make it a joke when they see you they say here is this one who was masturbating ((laughing)) so yha so I can say yes and no.”*

***Young men, Soweto, 19-21 years FGD***

However, when it came to participating in MMC trials, young men discussed how peers could potentially facilitate participation through peer pressure due to societal norms around circumcision. As Kagiso from Durban shared in disagreeing to another participant claiming that young men only get circumcised to protect themselves,

***Kagiso:*** “*What he is saying [that young men only get circumcised to protect themselves] is right but partially it is wrong. It says there is nothing that can cause someone to want to get circumcised. We as guys growing up there is something that can cause this guy for an example as he is not circumcised, we are all circumcised as guys, peer pressure is there.”*

***Young men, Durban, 16-18 years FGD***

##### Intimate partners

Although there was little discussion around intimate partners being barriers to participation directly, young women discussed whether or not they would want to be open with their partners about participation. This could be seen as an indirect barrier to participation if young women felt that they were unable to disclose participation to their intimate partners. Facilitators to participation were often motivated by distrust of partners or the in ability to use condoms at times due to intoxication, and the opportunity to access youth-controlled HIV prevention modalities.

Female participants discussed how their willingness to participate in research in which a vaginal swab was taken would depend on the gender of the person collecting the swab, especially if in a relationship. While some women expressed that they felt that they should be discussing participating with their partners, others felt that this would be problematic or unnecessary. For example, in response to another young woman claiming she wouldn't tell her partner about participating in an HIV prevention trial, Ayanda exclaimed:

***Ayanda**: “I disagree a little bit with her but some of it is correct but according to my opinion, like you have to sit down with him and tell him that you are doing this for the sake of your relationship because he is your partner. … Anything that is happening in your private part like you are sexually active you are [having] sex with him.”*

***Young women, Durban 16-18 years FGD***

However, others felt that they wouldn't want their partners to know that they were participating in research.

***Hope**: “I'll go for it but my partner I won't tell him because ((can't think of a reason)) ((laughing))”*

***Facilitator**: “So, you would keep it a secret from your partner?”*

***Hope**: “Yes, and if maybe neh, let's say he checks himself [gets tested] and he finds that he got it [HIV] ((laughing)) that means he got it somewhere, I will know my status isn't ? And he checked without knowing.”*

***Young women, Soweto, 22-24 years FGD***

Young women also discussed how they wouldn't tell their partners about participation in HIV trials because they would likely be misinformed and have false beliefs around what will happen to his partner while participating in the trial.

**Fancy Face:** “*You said what if they check you neh at the clinic? No, we won't go to the clinic, I'll tell him that isn't you know I'm attending a study here? No, my partner knows that I'm attending a study here. But I won't tell him about this vaccine, that I'm going to get false HIV cause I know he's crazy like, he's stubborn like, like he's mentality it's immature you see. So, I'll say like…I'll say they going to put false HIV [get the HIV vaccine] and then he goes get it elsewhere and then say I infected him, things like that”*

***Young women, Soweto 22-24 years FGD***

While other young women discussed that participating in research that allowed for more female-controlled methods could help to protect them and give them power and could also an opportunity for both partners to learn and be involved in research together.

***Mpho**: “I would take part neh? Our partners' neh? I can treat myself nicely but immediately when he goes out, I don't know he goes out and with who, his sneaking where. So I would take it for my safety like that, but anyway he knows, his also participating in the study that we both stable.”*

***Young women, Soweto, 16-18 years FGD***

Vaccine trials and PrEP were favored by women for access to female controlled discrete options to protect themselves against HIV when there is a lack of trust in the relationship (i.e., against unfaithfulness; when condoms weren't used). This is shown through this statement from Hope:

***Hope**: “Most of the time my partner doesn't use a condom. So because I don't know what he does, where he is, …I think if use it maybe when he comes he will find me strong [protected from HIV]… if he comes with the virus I won't get it, maybe I am strong”*

***Young women, Soweto, 19-24 years FGD***

Young men discussed how participating in biomedical HIV prevention research could help to build a better future for them and their future partners. Participating in this research may help to find a cure and in turn reduce the fear of acquiring HIV with a partner they might want to have children with.

***Sipho**: “Another thing that can make people participate is that, if you now know that okay there is something available as a cure for HIV most people will see life becoming easy because HIV is what causes life to be difficult because as youth we sometimes when we get old we become scared of having children cause the person you going to have a child with you do not know whether they are positive or negative unless if you do tests.”*

***Young men, Durban, 19-24 years FGD***

Young men also acknowledged that taking PrEP in a trial would be motivating as it could protect you from your partner who might be “doing something on the side.”

##### Family/Caregivers

Family members, and specifically caregivers, were also mainly considered as a barrier to participation. Some young women discussed how they were worried about their parents finding out about their participation, which resulted in some disagreement about the role that caregivers should play in young women's health decision-making. Overwhelmingly, young women feared that participating in HIV prevention research would ‘out’ them as sexually active to their parents, with varying consequences. For instance, Thanda explained:

***Thanda**: “So, now ja some can talk to you and stuff, but parents are different some can even chase you away from your home, so you see those things.”*

***Young women, Durban 16-18 years FGD***

This quote also highlights how for young women under 18 years of age, having to disclose to parents the criteria for participation may cause significant barriers for adolescent women under the age of consent. Even when participants may be old enough to consent to participate, barriers such as asking caregivers for transport money may also be a significant barrier to participation.

***Busi**: “Even though they would want to participate, because of certain reasons at home…Transport - the procedure of asking for money. Where do you say you are going? Parents will say we will take you there because you are still a teenager, still young, we are going to treat you like that and now you are doing things that are being done by adults. You will be judged also, things like that.”*

***Young women, Durban 19-24 years FGD***

Young women also feared that to parents, study participation would suggest risky sexual behavior and expressed concerns about a lack of privacy if asked to collect vaginal or seminal samples at home.

***Faith**: “Parents, naughty parents, who just dig in your bag and will find everything and ask now what are you doing no [talking and laughing] like there are no boundaries [participants laugh] no, but then like if your parent finds it they will find what they were looking for [laughs] what do you want.”*

***Young women, Durban 19-24 years FGD***

Participants in the same FGD spoke about the strong societal stigmatization around the sexuality of young women. This included some young women discussing how they may not want to participate in HIV prevention research that might disclose to their parents that they are sexually active, while others felt that young women need to take control of their own health.

***Thanda:*** “*I say no I'm thinking about my parent like what would she think when she finds out that I'm sexually active and stuff so, some wouldn't do it and ja.”*

***Ayanda:*** “*I think it's not about …what your parents think. It is your future, it's your life, you are the one who knows that now you are sexually active you need to, to do this and this to prevent myself from being you see, a laughing matter in the community.”*

***Young women, Durban, 16-18 FGD***

Due to societal norms around young women's gender roles and sexuality, young women expressed that communicating with parents was challenging. This is highlighted by a quote from Busi discussing the lack of open dialogue between young Black South Africans and their caregivers:

***Busi**: “As black children we are being discriminated that no, you can't do this thing they don't tell us why they[parents] just say don't do this.”*

***Young women, Durban 19-24 years FGD***

Young women aged 16–18 years old also highlighted a lack in parental communication skills regarding HIV prevention and family planning, which create barriers for young people to learn and have an open dialogue about risk prevention and in turn the ability to participate in research centered on preventing HIV.

***Londi**: “Some parents though, they do not judge by looking they wait maybe until they prove by themselves when that particular time comes but sooner or later your parent will find out. But then Ayanda is also right if your parent, because they are parents as Thanda has said, that they unable to sit down with their children that is why most children end up getting pregnant and stuff because a parent is scared to confront her child and tell the child that, you see my child if it is like this and this that means this is what is supposed to be happening”*

***Young Women, Durban, 16-18 years FGD***

#### Community

During discussions of participation in other HIV prevention research, young men expressed that social norms around circumcision and community-level views of altruistic actions, such as research participation, motivate young men to participate. For example, in the younger aged male FGD in Soweto, participants discussed how research participation could help young men “earn a name in the community for doing volunteer work.”

Other young men expressed potential fear in research participation, as it could cause conflict, and this could lead to violence in the community.

***Shakes**: “I would say no to being part of that group some people will discriminate against you, they'll judge you you see and call you names… So that now will make the heart sore. You see. And that can lead to conflict, then we end up fighting, stab each other, you know things between men never end.”*

***Young men, Soweto 16-18 years FGD***

While young men expressed concerns for being made fun of by their peers for procedural elements to participating in HIV prevention trials, young women feared that individuals in the community would perceive them to be living with HIV if they found out that they were participating in research.

***Gugu**: [In **response**to what barriers would prevent you from taking part in trials] “Being stereotyped by people. […] When they see you outside and seeing that you are taking medication, they might think that you are HIV positive maybe, ja that kind of stuff.”*

***Young women, Durban, 16-18 years FGD***

In order to resist some of societal level norms around sexual behavior and stigma attached to parents and other knowing about their sexual experience, young women discussed ways in which to mediate risks for participating in research (taking the power back into their own hands). For example, Ayanda in Durban shared,

***Ayanda**: “Like maybe let's say Zonke is my friend and I share lot of things with her and she will come and tell me that okay friend if it is like this you should do this and this like you don't have to really tell your parents if you don't feel comfortable. You can do your things like aside, in private.”*

***Young women, Durban 16-18 years FGD***

This also demonstrates the trust, community and friendship created among the participants in AYAZAZI.

#### Structural/Societal

##### Health care services and youth-friendly study environments

Participants discussed that interactions with health care providers in their community could often be challenging as their social networks are very interconnected, and they often know the health care providers personally. Young women expressed how this led to institutional distrust and anxiety around health care providers disclosing personal information to their parents.

***Thola**: “And what is painful is that the clinics, usually you can go to the ones that are around home ((local area)), so if a nurse sees you there and knows your parent you will be scared and end up maybe not doing all the tests that you went there for and say okay, ((what if / imagine)) I will do the pregnant test and if it comes out positive the first person that would know is this mother ((nurse)) and this mother will tell my mother.”*

***Young women, Durban 19-24 years FGD***

However, young women and men both discussed how participating in the AYAZAZI study allowed them to build trust, have more confidentiality and gain access to youth-friendly services. This was compared by participants to regular clinic visits where the lack of confidentiality was presented as a barrier to attending. As expressed by one young women 20–24 years from Soweto “*I came here because I didn't like to go to the clinic to check”*.

Distrust of health care provided in young women's communities was a common theme among young women in Durban as well, thus having the option of attending a clinic outside of their community that provided youth-friendly sexual health services facilitated young people to participate in our study.

***Noma**: “What made me love AYAZAZI the most is the fact that you always know your body like maybe if you have STIs as [mentioning note taker's name* ^*^ *] has mentioned that they first test you ((blood tests)), then they test you about yourself. So that thing makes me love AYAZAZI and also as they've already ((other participants)) said not going to the clinic in your community. What irritates me the most is that the nurses will pass the news, when they know each other they will keep chatting you see.”*

***Young women, Durban 19-24 years FGD***

Young men discussed how accessing care and services through research participation allowed for easier (less time consuming) access to sexual health services.

***Bheka:*** “*[another] thing if it is said that they should come like to the clinic, as my sister ((presenter)) was saying, like the process of the study. If they get to the clinic and find that they will maybe wait for like more than 3 hours, and that is what people don't like. So they want something that is quicker all the time. That is why I am saying that maybe it's 40% that would participate.”*

***Young men, Durban, 19-24 years FGD***

The strong sense of community and belonging through participation in AYAZAZI was emphasized by both young women and young men. And that this sense of community that AYAZAZI provided for youth was discussed as a strong motivator for participation in other youth-centered studies.

In Durban, young women discussed how they felt participating in AYAZAZI allowed for them to build friendship with other participants and felt supported by the study staff at AYAZAZI. Precious (age 16–18) exclaimed “The AYAZAZI team is so supportive I just love them all.” In Soweto the younger aged women also discussed how participating in AYAZAZI provided opportunity to build friendships and learn from other young people.

***Aphiwe**: “ey it's funny here hey man we get to know each other meeting different people yah”*

***Lena**: “okay, that's fine”*

***Kagiso**: “you get to learn about lot of things uhm get to know each other the the the groups opinions… yah”*

***Young women, Soweto 16-18years FGD***

#### Cross-Cutting Barriers and Facilitators

Social norms and gender inequities shaped willingness to participate in hypothetical biomedical HIV prevention research across all levels of the ecological model. For young men, societal level norms around the acceptance of male sexuality allowed for young men to be seen as altruistic and heroic for participating in research. While, at the same time young men feared being judged by peers for participating in research practices that were seen as unmasculine or shameful. For young women, societal level taboos around young women's sexuality created barriers for young women's participation in HIV prevention research which often requires young women to have had a history of sexual activity in order to be eligible or need parental consent if the participant is under the age of 18. Trust and distrust also operated on all levels to influence young women and men's willingness to participate in research. For example, mistrust of partners, parents, study procedures, skepticism around research evidence, and historical mistrust of health services due to negative encounters with health care providers were commonly discussed. Research participation willingness was facilitated by approaches that were youth-centered, and allowed for ease of access to information, services, support, care, trust, and a sense of belonging.

## Discussion

Our results of multi-leveled barriers and facilitators demonstrate that young men and women are interested in participating in biomedical HIV prevention research, but research programs need to be designed with their daily social realities in mind. Gender and power inequities affected youth's willingness to participate in biomedical HIV prevention trials, on individual, interpersonal, community, and structural levels. While distinct and gendered barriers and facilitators were noted within each level of the ecological model, societal gender norms and trust and mistrust cut across all levels to influence young women and men's willingness to participate in hypothetical biomedical HIV prevention research. Moreover, at each level young people's desire for youth-friendly sexual health services and information were noted by young women and men as key facilitators to participation.

### Societal Level Gender Norms

Adolescence is a transformative time period in which behavioral motivation is highly influenced by societal norms (19). Our results highlight how differences about whether to participate in hypothetical biomedical HIV prevention research are highly gendered and occur along societal expectations of what it means to be a young man or a young woman in South Africa. For young women, decisions were based largely on external factors including intimate partners, parents and societal perceptions of what participating in HIV prevention research would say about young women's sexuality and sexual behaviors. Whereas, for young men, decisions were more based on individual factors or peer-to-peer perceptions—specifically what other young men in their communities would think about them.

Healthy adolescent development includes increased decision-making capacity (19). In many global contexts, however, societal gender norms resist young women's individualism and decision-making power (33). One element of Connell's theory of gender and power is the gendered division of power, which is most often described on an interpersonal level between men and women in relationships. This conceptualization of power inequities within Connell's theory may be too narrowly focused on intimate relationship dynamics. Expanding this theory to other relationships including parents, peers and healthcare workers can help to conceptualize the complexity of power dynamics that young women must navigate in order to build their decision-making capacity across the life course (33, 34). Conversations that occurred among women in this study highlighted that navigating behavioral control and monitoring by parents and caregivers was one important barrier to research participation as well as accessing health care generally. The gender differences in parental perceptions and barriers to participation were contrary to a previous study conducted in Tanzania indicating that men were much more likely to decline participation in HIV vaccine trials due to parental concern for young men's health (33, 35–37). Parental control and monitoring has been previously discussed as a common occurrence for young women growing up in sub-Saharan Africa, and an important determinant of sexual behavior among adolescents and young adults in sub-Saharan Africa (33, 38, 39). Thus, while young men may face some parental discouragement once they've enrolled in the study, due to fear of their child's safety, parental control and monitoring may be more of a barrier for young women to enroll into a study in the first place. While some young women discussed the challenges of communicating choices to controlling parents, others navigated and resisted these controls by choosing not to communicate decisions around research participation. This may be a choice for young women wishing to participate in research who are able to consent for themselves, however remains a major hurdle for young women wishing to participate in research needing parental consent (40).

These findings highlight the gendered dynamic in sexual health studies among adolescents and young adults, whereby young women tend to receive ‘blame’ for adolescent pregnancy, HIV, etc., while young men rarely face this. Conversations within the FGDs reflect how young women experience shame and stigma regarding their sexuality, when sexual experience is required for research eligibility, whereas young men in the study were more likely to highlight that research participation would be viewed positively in their communities, potentially giving them more recognition, even allowing for them to increase their social stature. As young women move from childhood, through adolescence and into adulthood, they must navigate the different relationships they have been embedded within while they begin to form new relationships and make important decisions for themselves (41, 42). Although some discussions highlighted how young women navigated decisions regarding communicating research participation, societal pressures to conform to standards of femininity within young women's communities needs to be further acknowledged and understood (23, 43). The results from this study showcase how interacting relationships and societal pressures influence young women's willingness to participate in biomedical HIV prevention research, as well as their ability to communicate with partners, peers, and family about research participation.

### Distrust in Partners, Procedures, and Healthcare Professionals

Distrust was addressed by both young men and women, exploring personal relationships as well as perceptions of health care professionals, procedures and systems. These perceptions, however, were gendered. Young women discussed mistrust of their own intimate partners in two main ways. Some young women did not trust their partners enough to share with them about research engagement. Distrust of their partners was also presented as a motivator to participation, particularly if they perceived or suspected their partners to have other sexual partners. They recognized that their involvement in the study procedures could protect them from HIV and other STIs. These discussions are in line with much of the PrEP literature which centers on the ability for this HIV prevention modality to be more women-centered and controlled (44).

Our findings also highlighted young men's distrust in their own behaviors and their perceptions that PrEP could be useful in the short-term when engaging in activities (such as binge drinking) that may result in condomless sex and in turn increase the likelihood of acquiring HIV. While some research has examined the role of alcohol use on PrEP decision-making for young men who have sex with men (45, 46), there is limited research on motivations for PrEP use and PrEP implementation research participation among young men in heterosexual sexual relationships. Results further highlight the positive outlook on the future held by young men who were excited about the future implications that PrEP could have for starting a family in potentially sero-discordant partnerships.

In relation to institutional mistrust, young men were more likely to discuss their distrust for research procedures, particularly surrounding the collection of samples. These findings are in line with previous research conducted in the United States, highlighting high levels of medical distrust among young men living with HIV (47). Young women also discussed distrust of health care professionals due to issues of confidentiality and perceived judgements regarding young women's sexuality. This is further influenced by the discretionary power of health care workers, in which some women spoke about fearing health care workers will disclose information about STIs and sexual behaviors to their parents. Neglect for confidentiality is a common narrative among South African's interaction with the health care system (48). Across genders, young men and women discussed both real and potentially misinformed worries and skepticism regarding participation in biomedical HIV prevention trials. Misconceptions about trials procedures or effects influenced both young men and women's decision to participate as well as their decisions to discuss their participation with others. Similar to previous research highlighting the reasons for declining to participate in an HIV vaccine trial (33, 35–37), participants discussed how perceptions of the trials in the community, including the belief that an HIV vaccine could give a participant HIV or false-positive HIV status, would be influential in their decision to participate (49, 50). Thus, it is critical for young people to trust confidentiality processes and are provided with trusted, reliable, adequate and clear information about HIV vaccines and other biomedical modalities as well as both the benefits and potential side-effects from trial treatments.

### Young People's Desire for Youth-Friendly Services and Information

Overwhelmingly, young women and men in our study were willing to participate in HIV prevention trials, a finding similar to other studies conducted with youth in sub-Saharan African settings (49, 51). Youth had to weigh the benefits, access to youth-friendly services and information against fears of potential side-effects. Even after enrollment, previous studies have found that participants may drop-out due to fears about side effects (33, 35–37). Over the 12 months of follow-up in our cohort study, 95% of participants were retained in AYAZAZI. Although our study was not a biomedical trial, these results highlight how the desire for continuous access to appropriate, responsive, and youth-friendly sexual health services was strongly conveyed by young women and men in this study. Though it was a research study, AYAZAZI and the youth-engaged approach, provided a welcomed window into youth-friendly and youth-centered sexual health services, where they experienced positive, supportive relationships with staff and their peers. Young people may not feel comfortable discussing their sexual health with their family members, and often instead turn to their friends for advice and support (52). Research participation has provided opportunities for youth to learn more about HIV and ways in which they can protect themselves. Such opportunities, they noted were rarely provided at home, in the community, or within educational and health care institutions. Motivations for participation in hypothetical biomedical HIV prevention research were commonly centered on the desire to access youth friendly sexual health services and information, similar to the experiences they had while participating in AYAZAZI, highlighting that young people feel that participating in sexual health related research may be one of the few opportunities to gain access to these services (53–55).

### Recommendations

Many young women discussed how they find it challenging to discuss their sexual health with their parents and that participating in HIV prevention trials may disclose to their parents that they are sexually active, which would prevent them from participating. Results highlight the lack of parent-child communication among many adolescents and young adults in South Africa and their families (56, 57). Efforts are needed to engage with parents, guardians and caregivers to support them in learning how to effectively communicate with their children about sex and HIV, particularly in a setting with high STI and HIV prevalence. These efforts may include virtual tools that can support challenging child-parent conversations (58). However, in order for parents to be able to talk to their children about taboo topics like sexuality, they need opportunities to discuss their own experiences as young people and sexuality in general. Thus, efforts to engage parents and caregivers in dispelling myths, communicating accurate information and building a repertoire of vocabulary for which to use with their children are needed. Previous research with youth and caregivers in Soweto highlighted that within households without male caregivers, young women and men often take responsibility for their own health care, while also feel the need to educate caregivers on the importance of health promoting decisions (e.g., getting vaccinated for HPV) (17). Thus, parental supports may also include efforts aimed at bringing parents and caregivers of youth together to support one another and learn about emerging HIV prevention and other sexual health promotion technologies and programming. Finally, given the importance of peer relationships for youth, HIV research wishing to recruit young people should seek to enhance and build on peer-to-peer messaging.

While societal-level norms may make it easier for young men to participate in research, gender specific barriers, such as medical mistrust may prevent young men from engaging in research. Thus, efforts are needed to build trust with young men who have been historically disadvantaged by the health care system in South Africa (48, 59). Also, community-based programming aimed at shifting and transforming harmful gender norms and attitudes are critical for young women's agency in decision-making such as participation in research that is highly stigmatized in communities with engrained patriarchal hierarchies (60). Results from this study highlight the critical need to take into account gender and power relationships in designing HIV trials, and more broadly in responding to HIV prevention needs and youth friendly sexual and reproductive health services more generally. Given the successes in retaining and supporting participants through the youth-engaged approach undertaken by AYAZAZI, there is a critical need to improve and build upon existing youth-engaged approaches in order to successfully involve and retain young people in HIV research.

### Limitations

Focus groups were conducted among youth who were already participating in an HIV-related study that required biological sample collection, as such the views of these young people were likely more positive toward research participation than all South African youth. Thus, it is likely that the findings discussed herein cannot be generalized to all young trial naïve people in South Africa. Finally, in using FGDs while efforts were made to encourage and facilitate each individual to provide their opinions, given the group dynamic some participants may not have felt comfortable expressing their opinions.

## Conclusion

In order to enhance the efficacy, acceptability, and uptake of biomedical HIV prevention technologies there is a critical need to involve adolescents and young adults living at the epicenter of the HIV epidemic into research. Our study highlights that there are a plethora of barriers and facilitators that may impact youth's participation in biomedical HIV prevention research that run along gendered lines. This needs to be taken into account in designing future research and sexual and reproductive health programs. Societal gender norms, power dynamics and mistrust within intimate relationships, family households and society as a whole disproportionately affect young women's decision-making practices surrounding participation in research. Future research needs to take into consideration these lived realities and work toward involving families and creating youth-engaged research environments and opportunities which seek to redress inequitable gender relations.

## Data Availability Statement

The datasets presented in this article are not readily available because the de-identified data analyzed for this study cannot be shared publicly as we do not have community or REB approval to do so. Researchers and trainees who meet the required criteria can access data analyzed for this study through correspondence with the corresponding author Dr. Angela Kaida. In order to access data, researchers and trainees will need to be added as a researcher or trainee to the SFU research board application, an AYAZAZI data sharing and collaboration agreement. Requests to access the datasets should be directed to kangela@sfu.ca.

## Ethics Statement

The studies involving human participants were reviewed and approved by the Office of Research Ethics (ORE) of Simon Fraser University (Canada) and the Human Research Ethics Committee (HREC) of the University of the Witwatersrand (South Africa). Written informed consent to participate in this study was provided by participants aged 18–24 years and the participants' legal guardian/next of kin with participant assent for those aged 16–17.

## Author Contributions

KC, LL, JJD, and AK made substantial contribution to the design and conception of the work. JJD, MEB, JS, AK, PS, and SH supported data collection. LL, PS, SH, and KC conducted data analysis and interpretation. Project was supervised and supported by JJD, MEB, JS, MB, TN, GG, and AK. KC wrote the first draft of the manuscript. LL, SH, PS, JJD, MEB, JS, and AK wrote sections of the manuscript. All authors contributed to manuscript revision, read, and approved the submitted version.

## Conflict of Interest

The authors declare that the research was conducted in the absence of any commercial or financial relationships that could be construed as a potential conflict of interest.

## Footnotes

^1^In order to ensure that participants were able to pay for transport and were acknowledged for their time, AYAZAZI participants received small honorariums for participation, and were provided something to eat at each study visit.

^2^All names are pseudonyms chosen by youth.
